# Fitness Effect of the Isoniazid Resistance Mutation S315T of the Catalase-Peroxidase Enzyme KatG of *Mycobacterium tuberculosis*

**DOI:** 10.1093/gbe/evaf120

**Published:** 2025-06-23

**Authors:** Ugo Bastolla, Mikhail Rotkevich, Miguel Arenas, Manuel Arrayás, Marine Dogonadze, Anastasia Lavrova, Jorge Molina-Sejas, Michaël Tadesse, Ramón Xulvi-Brunet, Jonathan A G Cox, Dmitry Nerukh, Natalia González-Benítez, Michael Stich

**Affiliations:** Centro de Biología Molecular Severo Ochoa (CSIC-Universidad Autónoma de Madrid), Madrid, Spain; Centre for Genome Regulation, Barcelona, Spain; Department of Biochemistry, Genetics and Immunology and CINBIO, Universidade de Vigo, 36310 Vigo, Spain; Área de Electromagnetismo, Universidad Rey Juan Carlos, 28933 Móstoles, Spain; Saint-Petersburg State Research Institute of Phthisiopulmonology, Saint-Petersburg, Russia; Saint-Petersburg State University, Saint-Petersburg, Russia; Centro de Biología Molecular Severo Ochoa (CSIC-Universidad Autónoma de Madrid), Madrid, Spain; University of Warwick, Warwick, UK; Departamento de Física, Escuela Politécnica Nacional, Quito, Ecuador; Aston University, Birmingham, UK; Aston University, Birmingham, UK; Departamento de Biología y Geología, Física y Química Inorgánica, Universidad Rey Juan Carlos, 28933 Móstoles, Spain; Instituto de Investigación en Cambio Global (IICG-URJC), Universidad Rey Juan Carlos, 28933 Móstoles, Spain; Departamento de Matemática Aplicada, Ciencia y Tecnología de los Materiales y Tecnología Electrónica, Universidad Rey Juan Carlos, 28933 Móstoles, Spain

**Keywords:** *Mycobacterium tuberculosis*, antimicrobial resistance, isoniazid, S315T, oxidative stress, regularized maximum likelihood and minimum evolution (REGMLAME)

## Abstract

The mutation S315T of the catalase–peroxidase (CP) protein KatG of *Mycobacterium tuberculosis* is the most common mutation that confers resistance to the prodrug isoniazid. Here, we reconstruct its evolutionary history in 145 whole-genome sequences of *M. tuberculosis* from Russian hospitals, inferring 11 independent appearances of this mutation and 5 reversion events, with an estimated reversion rate 1,500 times higher than the rate of preserved nonsynonymous or intragenic mutations. This suggests that, contrary to the commonly held view, the mutation KatG(S315T) results in a fitness cost, possibly because of reduced tolerance to oxidative stress. Consistent with this interpretation, the mutant enzyme presents reduced CP activities. Applying the torsional network model (TNM), we found that the mutant protein shows more restricted thermal dynamics, although its functional site moves quite similarly to the wild type. Of the four internal clones where KatG(S315T) arose, two present high reproductive rates and secondary mutations at the 5′-UTR region of the gene encoding superoxide dismutase A (sodA), while the other two present significantly lower reproductive rates and lack mutations at genes related with tolerance to oxidative stress. Our results suggest that the resistance mutation KatG(S315T) incurs a fitness cost, which may be alleviated through compensatory mutations at the gene *sod*A or other genes that respond to oxidative stress, such as the previously known gene *ahp*C. This suggests that isoniazid treatment could be complemented with drugs that produce oxidative stress in order to hinder the propagation of resistant strains devoid of compensatory mutations.

Significance Isoniazid (INH) is a prodrug widely used for treating *Mycobacterium tuberculosis* infections that is activated by the bacterial catalase–peroxidase enzyme KatG. Mutations of this enzyme that abolish the activation result in resistance to INH, a serious public health problem, the most common being KatG(S315T), present in 74% of INH-resistant strains and considered to pose only mild fitness costs. Here, we reconstruct the evolutionary history of 145 wholly sequenced genomes of *M. tuberculosis* from Russian hospitals, inferring 11 KatG(S315T) mutation events and 5 reversions. The reversion rate, much higher than the rate of synonymous or intergenic mutations, strongly suggests that KatG(S315T) causes a fitness disadvantage in the absence of INH, possibly due to reduced resistance to oxidative stress. Of the four KatG(S315T) clones appearing at internal nodes, two had high reproductive rates and presented mutations at the promoter of the gene superoxide dismutase A (sodA), while the two clones without such mutations had low reproductive rates. This suggests that SodA can compensate for the reduced resistance to oxidative stress caused by KatG(S315T), and that INH treatment could be complemented with drugs that produce oxidative stress in order to hinder the propagation of resistant strains lacking compensatory mutations.

## Introduction

*Mycobacterium tuberculosis*, the causative agent of tuberculosis (TB), is responsible for the highest death toll due to a single pathogen in the world. To combat TB, a wide range of antimicrobial drugs have been developed and used extensively across the world for decades (World Health Organization 2023). Nevertheless, the emergence of multidrug-resistant strains poses a serious threat to the efforts in the fight against TB and represents a severe public health concern (Seung et al. 2015).

Here, we focus on the drug isoniazid (INH), used as an anti-TB drug since 1952. INH is one of the most widely used and most effective drugs against TB due to its high bactericidal activity, low cost, high bioavailability, excellent intracellular penetration, and narrow spectrum of action (Whitney and Wainberg 2002). INH is a prodrug that must be transformed into the isonicotinic acyl radical by the *M. tuberculosis* enzyme catalase–peroxidase (CP) KatG to become active (Johnsson and Schultz 1994). The clinically most common INH-resistance mutation is the S315T mutation of the catalase protein (Ramaswamy and Musser 1998) where serine 315 is replaced by a threonine. This mutation is present in 74% of the INH-resistant strains observed in 2019 (Valafar 2021), which reduces its ability to activate INH (Wengenack et al. 1998). This mutant was extensively reviewed by Unissa et al. (2016).

*Mycobacterium tuberculosis* has evolved different strategies against oxygen-free radicals (reactive oxygen species, ROS), both extracellular and intracellular, such as the mycobacterial cell wall, which serves as the first defense barrier against ROS. It also synthesizes antioxidant proteins, including the catalase KatG. This is a large dimeric protein with 744 amino acids with CP activity that uses heme as a cofactor. Its main physiological role is related to the protection against oxygen-free radicals during phagocytosis by human macrophages (Manca et al. 1999; Singh et al. 2004) and during bacterial respiration of nonfermentable substrates (Ng et al. 2004). Based on in vitro studies, on the crystal structure (Bertrand et al. 2004) and on molecular modeling studies, it was concluded that the S315T mutant presents conformational changes in the heme pocket that reduce the binding affinity of KatG for INH (Kapetanaki et al. 2003; Bertrand et al. 2004). This reduces its ability to oxidize INH (Bertrand et al. 2004; Ghiladi et al. 2004) and produces a strong decrease in INH activation (Zhao et al. 2006).

Regarding the CP activity, Wengenack et al. (1997) showed that the KatG(S315T) mutant presents a six and 2-fold relative reduction in the CP activity, respectively. Saint-Joanis et al. 1999) similarly found that KatG(S315T) has 50% lower CP activity than the wild type. Nevertheless, the idea that this mutant presents no fitness cost is prevalent in the field (Pym et al. 2002; Casali et al. 2014).

Here, we focus on the important S315T mutants of KatG using a database of 145 clinically characterized *M. tuberculosis* isolates collected from patients in Russia from 2007 to 2014 and subject to whole-genome sequencing, recently presented by Chernyaeva et al. (2020). A large fraction of these isolates (80%, corresponding to 116 genomes) present the KatG(S315T) mutation. We use this data to address the question of whether this mutation represents a fitness cost and, if it does, whether we can suggest candidate compensatory mutations. To this end, we inferred the phylogenetic tree of the 145 isolates using the genome sequence of *Mycobacterium canettii* as the outgroup, and we inferred ancestral sequences, thus reconstructing the complete history of all genomic mutations, including the key mutation KatG(S315T).

Interestingly, the four direct mutation events that we inferred at internal nodes of the tree had very different outcomes. Two of them had a large reproductive success, producing 108 and 7 sequences of the 145 isolate pool, while the other 2 produced only 2 and 4 descendants. We inquired whether we could characterize candidate compensatory mutations that can rationalize this difference. We reasoned that KatG(S315T) mutants may be more severely affected by oxidative stress, and we investigated genes related to the response to this stress.

We also investigated whether the wild type and the S315T mutant of the KatG protein, whose experimental structures are deposited in the Protein Data Bank (PDB), present different dynamical properties, as analyzed through an elastic network model (ENM) in the space of torsion angles that accurately reproduce the atomic fluctuations of the thermal dynamics of proteins (Mendez and Bastolla 2010).

## Results

### Inference of Phylogenetic Trees

We inferred phylogenetic trees using either amino acid or DNA substitution models, or a combination of both, and different sets of aligned sites.

For amino acids, our dataset consists of 6,458 polymorphic sites after removing 7 sites that correspond to known resistance mutations, including KatG(315), that are likely subject to strong positive selection. The program ModelTest-NG with the Bayes Information Criterion (BIC) criterion selected as best model the empirical substitution matrix STMTREV (Yang et al. 2014) with the options +F (fit of the stationary amino acid frequencies) and +G, which fits the across-site variation of the substitution rate with a gamma distribution (Yang 1993). The second-ranked model consists of the JTT matrix (Jones et al. 1992), again with the parameter-rich options +F +G. For nucleotides, our dataset consists of 13,332 polymorphic sites. The first-ranked model was the transversion model (TVM), which fits all parameters of the reversible substitution matrix with the only constraint being that the two transition rates from A to G and from T to C are equal, resulting in seven fitted parameters. The second-ranked model was the general time reversible (GTR) model, which is the most parameter-rich substitution model, with eight parameters. In both cases, the program selected the option +G.

We then used the program RAxML-NG (Kozlov et al. 2019) for inferring the maximum-likelihood (ML) phylogenetic trees of 16 combinations of data partitions and models. In the first group of models, we model nonsynonymous sites with amino acid substitution matrices and synonymous and intergenic sites with DNA substitution matrices. We either separated synonymous and intergenic sites into two different partitions (DNA2) or joined them. We considered different combinations of substitution models and different options. We imposed equal branch lengths for all partitions besides a scaling factor (option –brlen scaled in raxml-ng). The most parameter-rich models with 2 DNA and 1 protein partition fit 40 parameters. We name the resulting models and the inferred trees as follows:

1. AA_JTT_FG_DNA2_GTR_G
2. AA_JTT_FG_DNA_GTR_G
3. AA_JTT_FG_DNA_TVM_G
4. AA_JTT_F_DNA_GTR
5. AA_STMTREV_FG_DNA2_TVM_G
6. AA_STMTREV_FG_DNA_TVM_G
7. AA_STMTREV_FG_DNA_GTR_G
8. AA_STMTREV_F_DNA_TVM

In the second group of models, we only adopt amino acid substitution models, and consider either only variable amino acid sites or also synonymous sites, which allow assessing the impact of the invariant sites on our estimates of the parameters F and G. The models that we consider fit 20 parameters, except AA_STMTREV_F, which fits 19. We call these models as follows:

9. AA_JTT_FG
10. AA_STMTREV_FG
11. AA_STMTREV_F
12. AA + syn_JTT_FG
13. AA + syn_STMTREV_FG

Finally, we consider DNA-only models over the set of all variable sites. The resulting models, which fit either 10 (GTR + G) or 9 (TVM + G) parameters, were named as follows:

14. DNA_GTR_G
15. DNA_TVM_G
16. DNA_TVM

We compared the topologies of the 16 inferred phylogenetic trees using the Robinson–Foulds (RF) topological distance (Robinson and Foulds 1981). The trees determined from the same type of models are only moderately similar to each other (normalized RF distance from 0.20 to 0.40 for amino acid models, from 0.22 to 0.50 for DNA models, and from 0.10 to 0.38 for combined models). Trees determined from DNA models were rather different from trees determined from amino acid models (RF score between 0.44 and 0.66). The two most distant trees were those that did not share any site, determined from nonsynonymous sites and from DNA variation excluding nonsynonymous sites. Trees from combined models were more different from trees from amino acid models (RF score from 0.38 to 0.53) than from trees from DNA models (RF score from 0.17 to 0.57). These results indicate that the inferred tree topologies present extensive differences, particularly if they are determined with different types of models. In particular, the trees determined with the models AA_STRMTREV_FG and AAsyn_STMTREV_FG differ only by considering invariant amino acid sites, which affect the fitted value of the F parameter and the shape parameter of the gamma distribution. They present large differences (normalized RF distance = 0.33), which shows that adding invariant sites may have a substantial influence on the inferred tree.

For combined data types, the tree with the optimal (minimal) BIC score is the tree obtained with the model AA_STMTREV_FG_DNA2_TVM_G, with one amino acid partition and two DNA partitions. For DNA-only models, the BIC score selects as the optimal tree the tree obtained with the model DNA_TVM_G. For amino acid models, the optimal tree is AA + syn_JTT_FG. In order to compare scores obtained with different data, we normalized the log-likelihood (LL) scores and the BIC scores by dividing them by the product of the number of sites and the number of branches in the tree. Since DNA models assign higher probabilities because they have only four states, we also divided the scores by the maximum entropy of the substitution model, which is log(4) times the number of DNA sites plus log(20) times the number of amino acid sites. Combined, data achieved a lowest normalized BIC score of 0.0375, which is lower than the lowest normalized score of DNA data (0.0509), suggesting that the former are preferable. However, the normalized BIC score of amino-acid-only data is even lower: 0.03098 when only variable sites are considered and 0.0210 when synonymous sites are also considered.

### Ranking of the Tree Topologies with the REGMLAME Score

In the present section, we consider a novel criterion different from the BIC (and the ML on which the BIC is based) for ranking the inferred trees. Note that the BIC criterion cannot clearly distinguish between trees obtained with different types of data (DNA versus amino acids). In the next sections, we will consider six tree topologies and verify that the presented results are qualitatively robust with respect to the chosen tree. Therefore, the choice of the reference trees does not influence the results presented in the rest of the study, but we think that it is interesting from the point of view of methods for phylogenetic inference.

Since the seminal proposal by Felsenstein (1981), the ML criterion has the most adopted method for inferring phylogenetic trees. The likelihood score must be corrected with an appropriate penalization of the number of fitted parameters through the AIC or BIC score. This approach has been enormously influential in developing quantitative phylogenetic methods and brought a tremendous advance in the field. However, despite previous hope that the ML method is unbiased, simulation studies indicated that it can be biased, i.e. it can consistently reconstruct a wrong tree even in the limit of infinite data (Kuhner and Felsenstein 1994).

It is not difficult to identify one source of this bias. The phylogenetic LL score consists of the logarithm of the probability of the data given the tree, which is computed as the elements of the matrix $e^{tQ}$ where *Q* is the substitution matrix and *t* denotes the length of the branch of the phylogenetic tree (time). Therefore, the LL is expected to be strongly correlated with the fitted parameter *t*, with a positive correlation for branches where there are many substitutions and a negative correlation for those with few substitutions. In accordance, we observed that the correlation is strong and positive in data obtained from distantly related proteins (Lorca-Alonso et al. 2025). For the present data, which consists of closely related sequences, we found a negative correlation between mean branch lengths and LL scores.

**Fig. 1.**
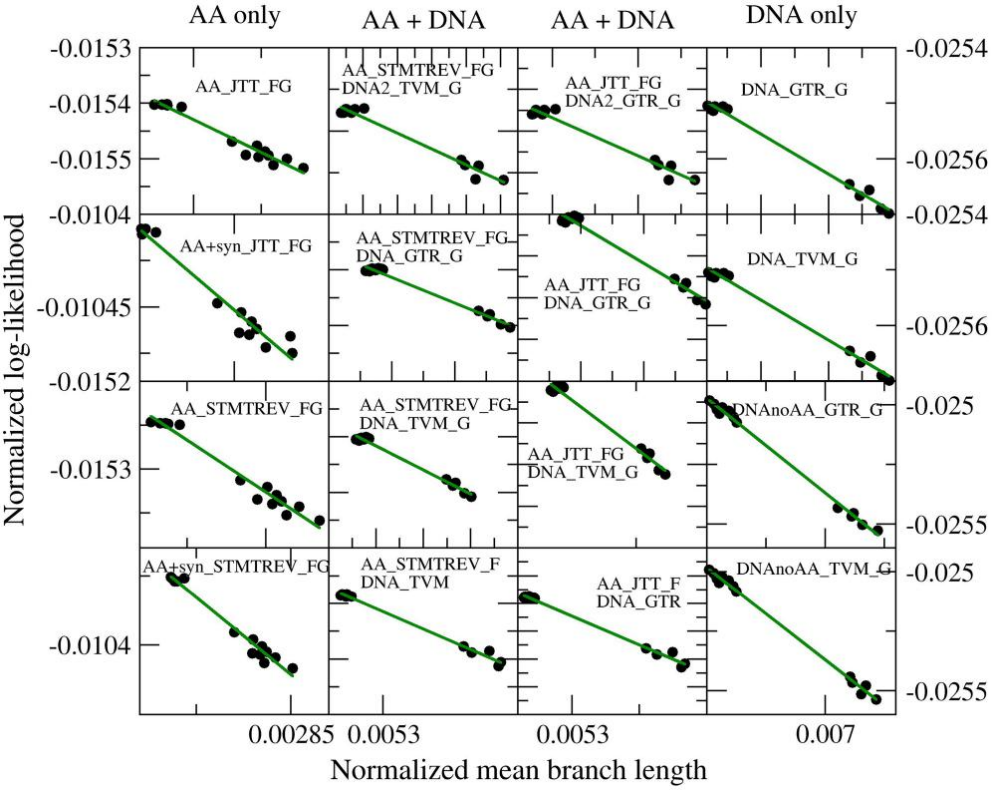
Relationship between normalized log-likelihood score and mean branch length for the sixteen inferred three topologies and branch lengths fitted with different substitution models. First column: amino-acid models JTT and STMTREV. They provide higher likelihood and shorter branch lengths to all considered topologies than the nucleotide-based models GTR and TVM (fourth column). Combined models (amino-acids and DNA) are shown in the second and third column and they provide intermediate branch lengths and log-likelihood. Note that all models show strong negative correlation between the log-likelihood and branch lengths.

Figure 1 shows the normalized LL score versus the mean-fitted branch length. Each plot is based on one of the combinations of models and partitions listed above, plus the DNA model DNAnoAA, which excludes nonsynonymous sites, which tend to evolve at different rates from synonymous and intergenic sites. Each point represents 1 of the 16 inferred phylogenetic trees. We fitted their branch lengths by maximizing the LL score with the given model with the program RAxML-NG (Kozlov et al. 2019). The nucleotide-based models (Fig. 1, right column) generated much lower normalized LL scores than the amino-acid-based models (left column), while combined models achieved intermediate scores.

The main result of this analysis is that the correlation between LL and mean branch length is extremely strong (correlation coefficients ≤−0.95 in all cases, excluding the trees inferred with the data DNAnoAA, which present very low likelihoods despite short branches and are the most different from the other trees, so they appear to have low quality). This means that 90% of the variance of the LL score depends on the value of the fitted branch lengths. In the context of regression, it is strongly recommended to regularize the score of the regression (here the LL) by imposing a penalty on large or, in this case, low values of the fitted parameters (here $t_{a}$), for instance, through the Tikhonov regularization (also known as ridge regression). The very strong correlation that we observe here between the score of the fit (LL) and the fitted parameters ($t_{a}$) strongly supports the regularization procedure. Regularized fits are much more accurate than nonregularized ones, which can produce even nonphysical fitted parameters (wrong sign). The regularized score is analogous to free energy, while nonregularized regressions only consider energy (Dehouck and Bastolla 2017). Lorca-Alonso et al. (2025) proposed a regularized fit that minimizes the sum of the negative LL plus the sum of branch lengths multiplied by the regularization parameter Λ. We called this score the “regularized ML and minimum evolution” (REGMLAME) score:


(1) $$\mathrm{REGMLAME}=-LL(t_{a})+\Lambda \underset{a}{\sum }\;t_{a}\;$$

Although there is currently no software that performs the regularized fit of the branch lengths $t_{a}$, it is possible to determine approximately the REGMLAME score a posteriori for a given tree topology and branch lengths fitted with a given substitution model through the following procedure applied to any given substitution model. (i) We fit $t_{a}$ through ML (Λ=0) for all tree topologies using the same model with the program RAxML-NG (Kozlov et al. 2019). Through this procedure, we determined the data plotted in Fig. 1. (ii) We determine Λ by fitting the LL score as a function of the sum of branch lengths across all trees. (iii) We compute the REGMLAME score using Equation (1), which is equivalent to correcting the LL by subtracting the value expected given the branch lengths. We report in supplementary table S1, Supplementary Material online for all 16 tree topologies the mean, standard error of the mean (SEM), and coefficient of variation of the REGMLAME score, the BIC score, the branch length, and the ranking obtained with these scores across all substitution models.

**Fig. 2.**
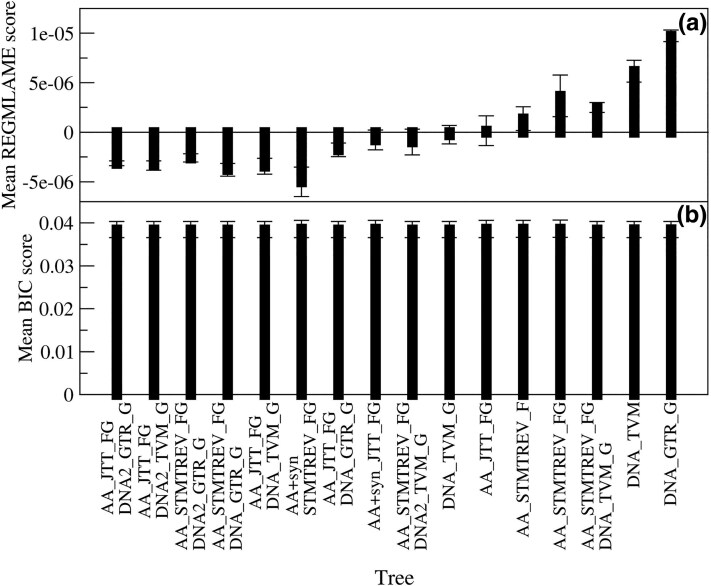
For each of the 16 tested tree topologies, we show the mean across all models of the REGMLAME score (a) and the BIC score (b). The error bars represent the SEM. We rank the trees according to the ratio between the mean and SEM of the REGMLAME score.

Unlike the LL and BIC scores, the REGMLAME score does not correlate with the mean branch length. Figure 2a shows the mean of the REGMLAME score for different trees. We ranked the trees according to the ratio between the mean and the SEM of the REGMLAME score. This ranking selects as best tree the tree AA_JTT_FG_DNA2_GTR_G, which has low REGMLAME scores for all types of substitution models, including combined models, amino-acid-only models, and DNA-only models (Fig. 2a). Other trees also have significantly negative REGMLAME scores, which means that their LL is higher than expected given the fitted branch lengths for most of the considered models. In contrast, the mean BIC score is not significantly different across different trees (Fig. 2b). In supplementary fig. S1, Supplementary Material online, we show, for each tree topology, the minimum BIC score (a to c) and the minimum REGMLAME score (d to -f) across the three types of substitution models that we considered: combined models (supplementary fig. S1a and d, Supplementary Material online), AA-only models (supplementary fig. S1b and e, Supplementary Material online) and DNA-only models (supplementary fig. S1c and f, Supplementary Material online). Trees inferred using combined models tend to have good BIC scores also for DNA-only models and the other way round, but they have poor BIC scores for AA-only models, while trees inferred with AA-only models have relatively poor BIC scores with other types of models. In contrast, some trees have good (negative) REGMLAME scores for all types of models because the decrease in LL is compensated by the increase in branch lengths (supplementary fig. S1, Supplementary Material online, plots d, e, f).

We will analyze in the rest of the study six trees that have low REGMLAME scores across all types of models. They are: (i) AA_JTT_FG_DNA2_GTR_G; (ii) AA_STMTREV_FG_DNA_GTR_G; (iii) AAsyn_STMTREV_FG; (iv) AA_JTT_FG_DNA_TVM_G; (v) AA_STMTREV_FG_DNA2_TVM_G; and (vi) DNA_TVM_G. These trees span a large diversity of tree topologies, and we inferred them using all types of models (combined, amino-acid-only, and DNA-only). The reported results are remarkably robust for each of them.

### Ancestral Sequence Reconstruction and Rates of Direct and Reverse Mutations

**Fig. 3.**
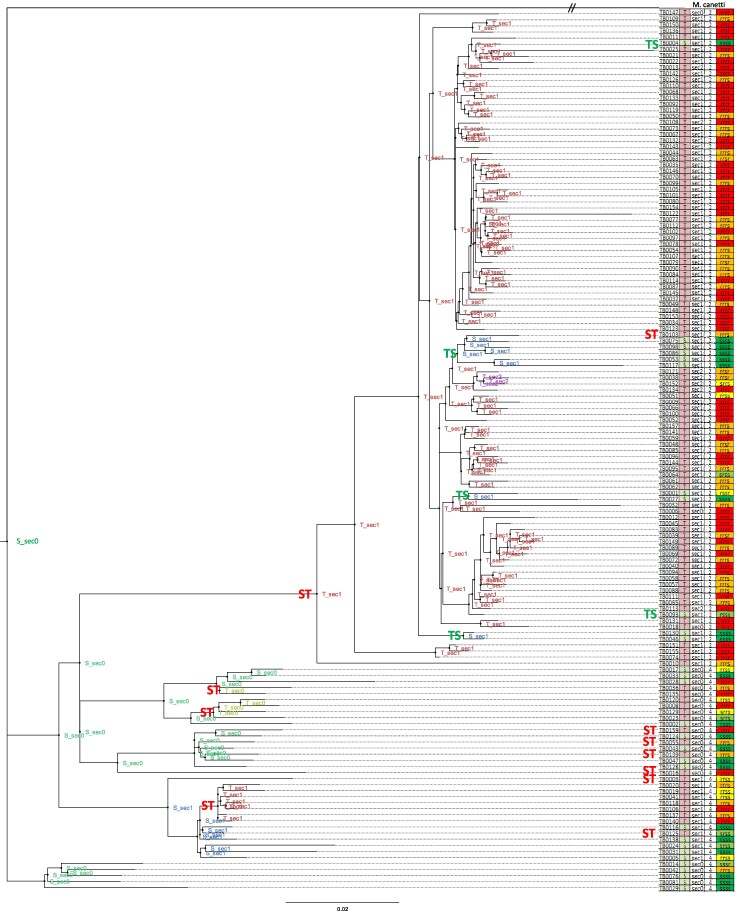
Inferred phylogenetic tree and reconstructed ancestral sequences. For each ancestral sequence at internal nodes, we indicate whether the residue at site KatG(315) is S or T and the number of secondary mutations of genes related to oxidative stress. We denote the events that produced the first occurrence of the resistance mutations or its reversion to S315 as ST (red) and TS (green), respectively. For each extant sequence (leaf), we indicate on the right the identity of the residue KatG(315), the number of secondary mutations, the lineage (either two or four for all of the sequenced strains) and the resistance (*r*) or susceptibility (*s*) to the four drugs Streptomycin, INH, Rifampicin, and Ethionamide. The red color indicates multi-resistant strains, green means susceptible, and the other colors indicate conditionally susceptible strains. The internal node labels indicate the amino acid at position 315 (either S or T) and the number of secondary mutations at genes involved with oxidative stress (0, 1, or 2). Since the label is difficult to read, we differentiate them based on color: dark green (S_sec0), blue (S_sec1), light green (T_sec0), red (T_sec1), and purple (T_sec2).

We plot in Fig. 3 the reference phylogenetic tree AA_JTT_FG_DNA2_GTR_G. One can see that the tree separates well sequences that belong to two main phylogenetic lineages, lineage 2 and lineage 4, which supports its overall validity.

For each of the six selected tree topologies, we determined ancestral sequences and branch lengths with the program RAxML-NG, applying the substitution models STMTREV + F + G for amino acid data and TVM + G for nucleotide data. We also tested that the results remain qualitatively robust when we apply the second-ranked models, JTT + F + G and GTR + G, respectively, for determining branch lengths and performing ancestral sequence reconstruction (ASR).

We counted the number of mutation events inferred at each node for each genomic site. We grouped mutations into five sets according to the sites where mutations lead to amino acid changes:

(A1) The direct resistance mutation KatG(S315T)
(A2) The reversion mutation KatG(T315S)
(A3) All other amino acid mutations known to be related to antimicrobial drug resistance (res) (supplementary table S2, Supplementary Material online)
(A4) Amino acid mutations related to resistance to oxidative stress (sec) (supplementary table S2, Supplementary Material online), which we consider as candidate secondary adaptations to overcome the possible decreased function of KatG
(A5) All other amino acid mutations

We considered four sets of sites where mutations do not lead to amino acid changes:

(S1) Synonymous mutations or mutations at 5′-UTR regions of genes related to resistance
(S2) Synonymous mutations or mutations at 5′-UTR regions of genes related to oxidative stress (sec)
(S3) All other synonymous mutations
(S4) All other mutations at intergenic sites

We did not find in our data any amino acid mutation at genes involved in oxidative stress (A4) or any intergenic or synonymous mutation at genes involved in resistance (S1). We indicate at each internal node of the tree in Fig. 3 the amino acid at site KatG(315) (either S, which is the wild type, or T, which is the resistant variant) and secondary resistance genes (sec, this only includes mutations at 5′-UTR regions) with respect to the root node of the tree.

**Fig. 4.**
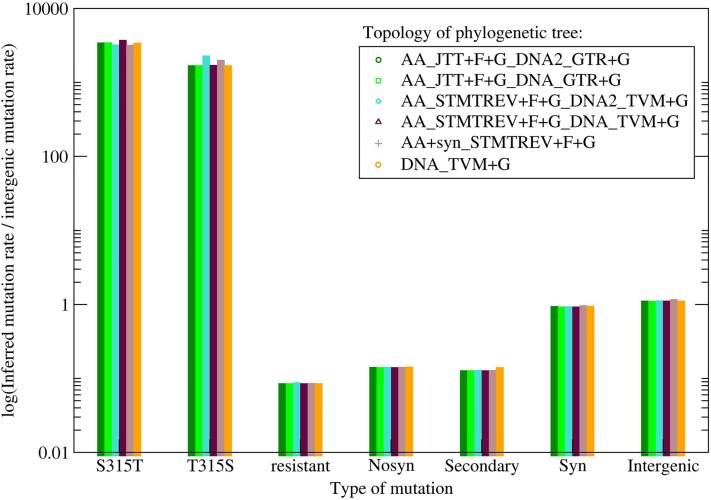
Relative rates of various types of preserved mutations inferred by adopting six different topologies of the phylogenetic tree. From left to right, the shown mutations are: (i) the resistance mutation KatG(S315T); (ii) its reversion KatG(T315S); (iii) other amino acid mutations known to confer antimicrobial resistance; (iv) nonsynonymous mutations at other genes; (v) synonymous mutations and mutations at the 5′-UTR regulatory region of genes known to be related with resistance to the oxidative stress, which we denote as secondary mutations; (vi) synonymous mutations at other genes; and (vii) mutations at integenic regions of other genes, which we adopt for normalizing the rates.

For each type of mutation and each tree topology, we inferred their rates by dividing the inferred number of mutations by the total number of possible mutations of that type times the sum of branch lengths. Note that these rates do not represent the bare mutation rate, since these mutations occurred long enough in the past to experience both negative and positive selection. Except for KatG(S315T), they did not become the majority, so the rates do not represent substitution rates either. They are akin to polymorphisms, but our evolutionary analysis allows us to assess reversions, which would not be possible with a simple analysis of polymorphisms. To avoid misunderstandings, we refer to these rates as “rates of preserved mutations.” The seven types of inferred rates of preserved mutations are represented in Fig. 4 for each of the studied tree topologies. They present clear signs of negative selection. Excluding the resistance mutation KatG(S315T), intergenic sites presented the highest rate, immediately followed by synonymous sites, while nonsynonymous sites presented a lower rate (nonsynonymous-to-synonymous ratio = 0.15). Mutations at known resistance genes and genes related to oxidative stress (secondary mutations) presented a rate even lower than the generic nonsynonymous rate.

The resistance mutation KatG(S315T) presented the highest of all rates. For all 6 tree topologies, we inferred 4 independent mutation events in KatG(S315T) before internal nodes and 6 or 7 mutation events before leaves, resulting in a total of 10 to 12 direct mutations. Surprisingly, we inferred a high number of KatG(T315S) reversions (from five to seven) for all tree topologies (Table 1). The corresponding rate of preserved mutations is >1,000 times higher than the neutral baseline provided by intergenic mutations (Fig. 3), strongly suggesting that positive selection is acting to enhance the KatG(T315S) reversion almost as much as the resistance mutation KatG(S315T), although the reversion may cause the loss of INH resistance.

**Table 1:** Results for all six selected phylogenetic trees based on the two-sample, one-tail *t*-test with 2 degrees of freedom

Tree	Direct S315T Internal nodes, leaves	Reversions T315S Internal nodes, leaves	Offspring Internal nodes, sodA mutation	Offspring Internal nodes, no sodA mutation	*t*(diff.repr.rates)	*P*-value
AA__JTT_FG DNA2_GTR_G	4, 7	3, 2	108, 7	2, 4	3.20	0.025
AA _JTT_FG DNA_GTR_G	4, 7	3, 2	108, 7	2, 4	3.19	0.025
AA_STMTREV_FG DNA2_TVM_G	4, 6	4, 3	108, 7	2, 4	3.08	0.027
AA_STMTREV_FG DNA2_TVM_G	5, 7	3, 2	108, 7	2, 4	3.44	0.021
AA + syn_STMTREV_FG	4, 6	4, 2	108, 7	2, 4	3.78	0.016
DNA_ TVM_G	4, 7	3, 2	108, 7	2, 4	3.05	0.028

First column: examined phylogenetic tree. Second and third columns: number of direct mutations KatG(S315T) and reversions KatG(T315S) before internal nodes and leaves of the tree. Fourth and fifth column: number of descendants of the internal nodes that possess or not a mutation at the 5′-UTR region of sodA. Sixth and seventh column: Student’s *t*-value and *P*-value (2 degrees of freedom, 1 tail) of the difference between the mean reproductive rates of the groups of mutant clones that do and do not present a mutation at the sodA gene for every examined tree.

### Study of KatG Dynamics with the TNM

The fact that the reversion KatG(T315S) appears to be positively selected (the nonsynonymous-to-synonymous rate ratio is much larger than one) suggests that the direct mutation incurs a fitness cost. This finding is consistent with previous reports that the KatG(S315T) mutant exhibits a 6- and 2-fold relative reduction in CP activity, respectively (Wengenack et al. 1997). To investigate this issue further, we predicted the atomic fluctuations of the KatG protein in the native state with the TNM (Mendez and Bastolla 2010), using both the structure of the wild-type protein (PDB code: 2cca) and the mutant S315T (PDB code: 2ccd). The root mean square deviation (RMSD) of 0.33 Å indicates the high degree of similarity between the two structures at the single-chain level; however, at the dimer level, the structures differ significantly, as evidenced by an RMSD of 38.8 Å.

### Prediction of Atomic Fluctuations

**Fig. 5.**
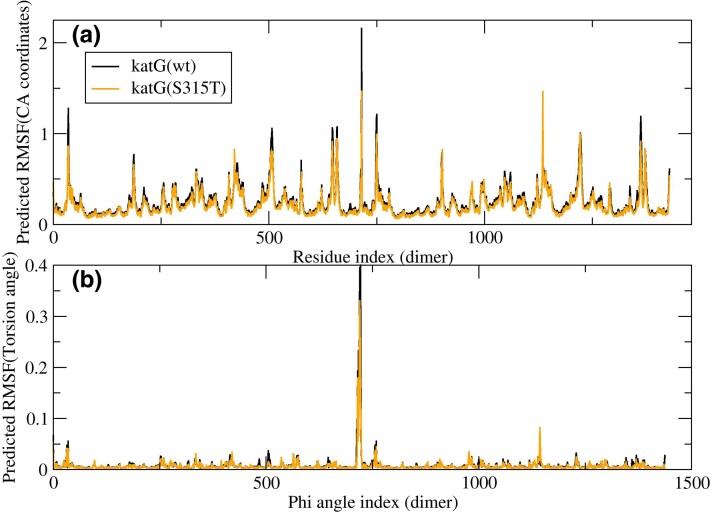
Predicted atomic fluctuations (a) and torsion angle fluctuations b) of the wild-type protein KatG (black) and the S315T mutant (soft lines).

We show in Fig. 5a the predicted atomic fluctuations for all residues of the dimer, and in Fig. 5b, we show the fluctuations of the torsion angles. In both cases, the wild-type protein (black lines) exhibits larger predicted fluctuations than the mutant protein (soft lines, which are always smaller than or equal to the black lines). Globally, the root mean square fluctuations are 1.19 Å for the wild type and 1.06 Å for the mutant. These differences are small (10%), and it is not clear whether these reduced fluctuations can account for the reduced catalytic activity.

### Predicted Dynamical Couplings

**Fig. 6.**
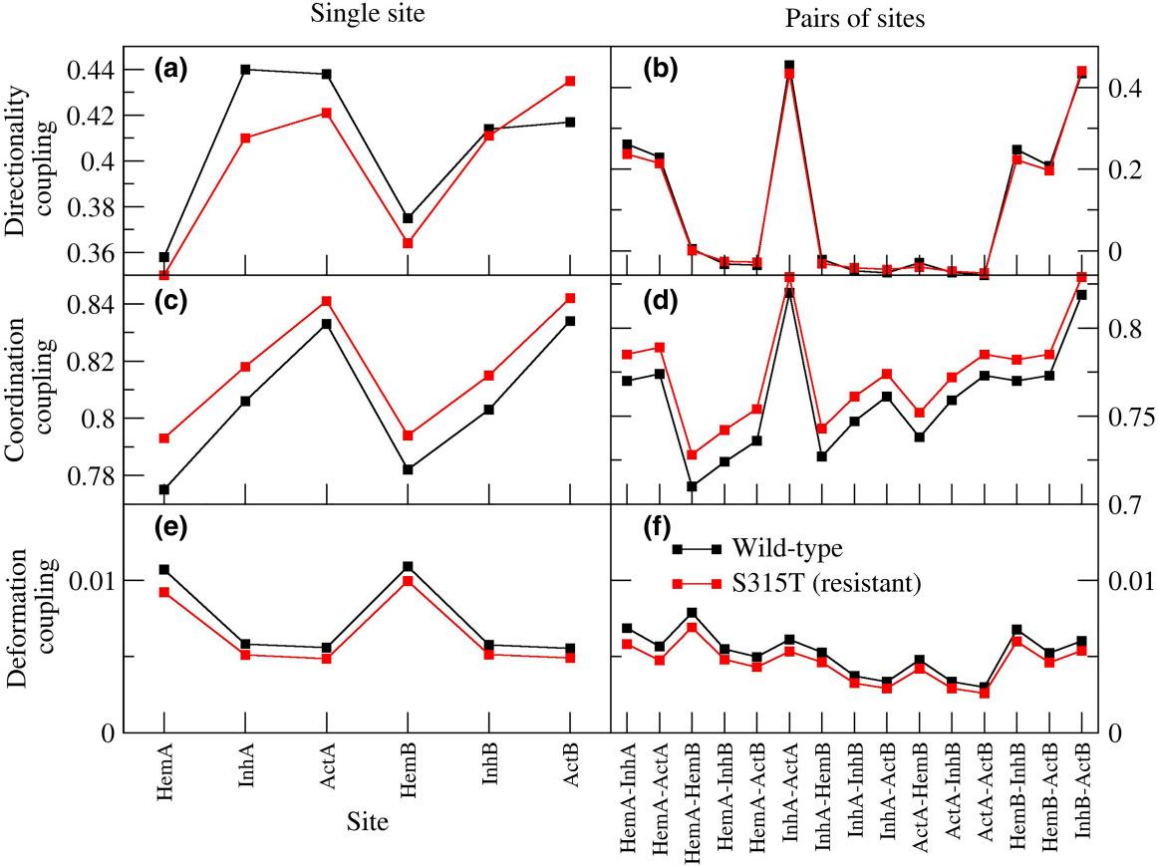
Predicted dynamical couplings within (a, c, e) and between (b, d, f) functional sites of the wild-type protein KatG (black) and the S315T mutant (soft lines).

We also measured the dynamical couplings between the three functional sites (heme-binding site, INH-binding site, and active site) of the two chains of the protein (Fig. 6). Atoms at the INH-binding site and the active site of both protein chains tend to move co-directionally (Fig. 5a), their interatomic distances exhibit minimal fluctuations (Fig. 6c), and there is no significant deformation of one site by the other (Fig. 6e). The heme-binding site is the more dynamic from all points of view, possibly because it is larger. The INH site and the active site in the same chain are strongly coupled to each other, whereas sites at different chains are less coupled (Fig. 6b, d, and f). The couplings of the mutant protein (red) are nearly identical to those of the wild-type protein (black), indicating that this analysis does not reveal any significant differences in the native dynamics of these proteins, aside from the reduced fluctuations observed in the mutant.

### Candidate Compensatory Mutations

With the six selected trees, we inferred four events in which the mutation KatG(S315T) appears for the first time at an internal node of the tree. The corresponding clones presented very different fates. For the reference tree presented in Fig. 3, in two cases, the mutation produced a large number of offspring (7 and 108), while in the remaining two cases, the number of offspring was very small (2 and 4). We show in Table 1 results for all six selected trees. We reasoned that this low reproductive rate may stem from the reduced ability of the mutant to cope with oxidative stress, as supported by the lower catalytic activity of KatG(S315T) mutants (Wengenack et al. 1997) and by our observation that KatG(T315S) reversions seem to be positively selected. In contrast, the high reproductive rate of the other mutants might have been favored by secondary mutations that increase their ability to withstand oxidative stress. Therefore, we looked for such secondary adaptations in the form of mutations in genes and regulatory regions of genes related to oxidative stress (supplementary table S2, Supplementary Material online). We did find eight such mutations, six at the 5′-UTR region of the gene encoding superoxide dismutase A (sodA), one at the 5′-UTR region of katG itself, and one at the 5′-UTR region of furA, which is a negative regulator of katG. However, only two mutations at the 5′-UTR region of sodA at positions −719 (G to C) and −654 (A to C) co-occurred with the KatG(S315T) mutation.

**Fig. 7.**
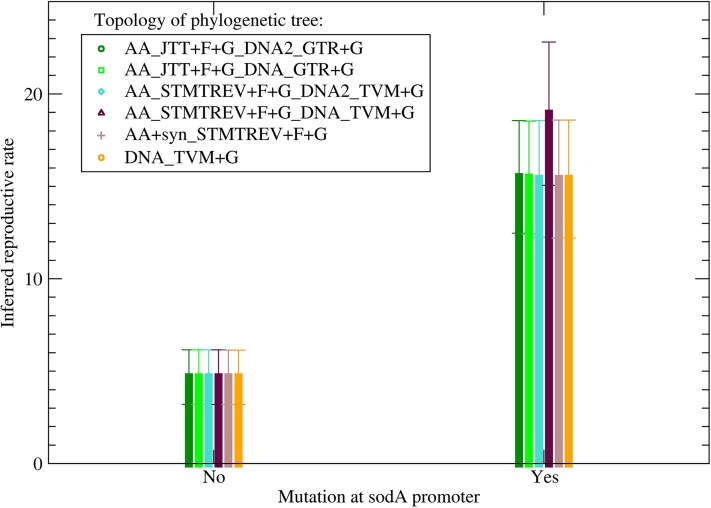
Mean and standard error of the mean of the reproductive rate of ancestral sequences of the mutant KatG(S315T) that did not present (left) and did present (right) mutations at the 5′-UTR region of the superoxide dismutase gene sodA.

We grouped the four mutants where katG(S315T) first appeared at some internal node in two groups, one with and one without mutations at the 5′-UTR region of the sodA gene. We measured the reproductive rate of each clone *c* as the logarithm of its number of offspring $n_{c}$ divided by the estimated time from the origin of the clone to the present time $\tau_{c}$. To estimate the time $\tau_{c}$, we measured the time $t_{0c}$ from the root of the phylogenetic tree to the given clone and the average time $t_{cp}$ from the clone to present-day sequences, expressed in units of number of substitutions per site. We chose the time from the root of the phylogenetic tree to the present as the time unit. We then estimated the time elapsed from the origin of the clone to the present as $\tau_{c}=t_{cp}/(t_{cp}+t_{0c})$, obtaining the reproductive rate as $r_{c}=\log\;(n_{c})/\tau_{c}$. Figure 7 shows the mean and the SEM of $r_{c}$ for the two groups of clones across each phylogenetic tree on which the inference is based. We show in Table 1 the probability that the two mean reproductive rates are equal, based on the two-sample, one-tail *t*-test with 2 degrees of freedom, for each phylogenetic tree. We can reject the null hypothesis with a probability between 1.6% and 2.8% for all six trees.

## Discussion

The CP mutation KatG(S315T) is the most prominent mutation by which *M. tuberculosis* develops resistance to the antimicrobial drug INH, since it fails to activate INH. In our dataset, 80% of the genomes presented this mutation.

### High Rate of Reversions KatG(T315T) Suggests a Fitness Cost

We studied the history of this mutation and other mutations in our dataset through phylogenetic tree inference and ASR (Fig. 3). Note that the examined mutations survived for a long time after their appearance, experiencing both negative and positive selection, which is reflected in their rates, which we denote as rates of preserved mutations. We found a very high rate of KatG(S315T) mutations compared with the neutral rate of synonymous mutations and mutations at intergenic sites (the ratio is more than a thousand times higher; see Fig. 3), indicative of positive selection. Surprisingly, however, we also observed a very large reversion rate (1,600 times higher than the intergenic rate, Fig. 4). This fact strongly suggests that positive selection favors the KatG(T315S) reversions, contrary to the hypothesis that the KatG(S315T) mutation has a very low fitness cost (Pym et al. 2002; Kapetanaki et al. 2005; Hazbón et al. 2006; Casali et al. 2014). Our conclusion is consistent with the observation that the KatG(S315T) mutant presents a 6- and 2-fold relative reduction in CP activity, respectively (Wengenack et al. 1997). This result is also in line with work that demonstrated that high mutation rates increase the probability of reversion toward the wild type when compensatory mutations do not fully restore the fitness costs (Pennings et al. 2022).

These results are also reminiscent of the proposed but disputed relationship between catalase activity and virulence. Middlebrook and Cohn (1953) showed that INH-resistant isolates of *M. tuberculosis* that lack catalase activity are attenuated in guinea pigs. They suggested that since KatG is required for protection against free oxygen radicals within the macrophages, it must be considered as a virulence factor (Middlebrook and Cohn 1953; Wilson et al. 1995; Li et al. 1998; Pym et al. 2002; Cardona et al. 2003; Ng et al. 2004). In contrast, other studies in mice and guinea pigs showed no correlation between loss of KatG activity and virulence of *M. tuberculosis* ( Pym et al. 2002).

The inferred evolutionary histories, and in particular, the reversions of the resistance mutation KatG(S315T), depend on the choice of the outgroup species. For this purpose, we chose *M. canettii* due to its unique evolutionary position, which is well recognized in the literature as optimal for rooting the *M. tuberculosis* complex (Brosch et al. 2002), since it is its closest known relative, representing an early-diverging lineage that allows for accurate inference of evolutionary relationships (Comas et al. 2013; Ngabonziza et al. 2020; Chiner-Oms et al. 2022). Moreover, *M. canettii* strains have common phenotypic and biochemical characteristics that clearly differentiate them from the rest of the *M. tuberculosis* complex (Fabre et al. 2010).

### Comparison of the Predicted Dynamics of the Wild-Type and Mutant Protein

We investigated the native dynamics of the wild-type and mutant enzyme using an ENM (i.e. the harmonic approximation of the structure-based model of the protein) in the torsion angle space (Mendez and Bastolla 2010). We found very similar dynamical couplings (Alfayate et al. 2019) between the functional sites (heme site, INH-binding site, and active site) of the wild-type and the mutant protein (Fig. 6), indicating that the native dynamics are not disrupted by the KatG(S315T) mutation. However, the mutant protein presents reduced dynamical fluctuations (Fig. 5), which is consistent with its diminished catalytic activity.

### Compensatory Mutations Enhance the Reproductive Rate

An interesting observation that arises from our evolutionary reconstruction is that the two mutants where the S315T mutation arises at an internal node of the tree undergo quite different fates. Two of them produced many offspring (7 and 108), whereas the remaining two produced few offspring. We investigated whether this difference may be attributed to any secondary mutation that might have compensated for the reduced oxidative stress tolerance associated with KatG(S315T). Our analysis focused on mutations in genes and regulatory regions related to oxidative stress resistance.

We identified two such mutations at the 5′-UTR region of the gene sodA (positions −719 and −654, respectively) that codes the Mn-containing protein SodA. SodA is a protein that relies on the accessory secretion factor SecA2 for secretion and plays a key role in the virulence mechanism of *M. tuberculosis*, enabling the bacteria to elude the oxidative attack of macrophages (Braunstein et al. 2003). Additionally, SodA with two other proteins, DoxX and SseA, forms a membrane-associated oxidoreductase complex (MRC) that functions in detoxification. Disruption of any MRC component leads to the accumulation of cellular oxidative damage, ultimately resulting in nonviable bacteria (Nambi et al. 2015). SodA is subject to complex regulation, likely involving transcriptional activators and autoregulatory mechanisms, where the SodA protein itself modulates its own synthesis in response to its intracellular levels (Fee 1991). Typically, SodA proteins, which protect cells from oxidative stress, are produced only when the cell detects oxidative damage, as their expression requires induction. However, mutations at the regulatory region may lead to constitutive expression of these protective proteins (Greenberg et al. 1990).

One of the two mutations at the *sod*A 5′-UTR region that we identified was present in each of the two ancestral sequences with many offspring but both were absent in those with fewer offspring. The significant difference in the estimated mean reproductive rates of the two groups of sequences (Fig. 7 and Table 1) suggests that these mutations may provide a fitness advantage by compensating for oxidative stress, possibly producing the constitutive expression of SodA and conferring increased resistance.

Note that superoxides had been previously linked to the action of the drug INH. Wang et al. (1998) observed that endogenous superoxide contributes to intracellular activation of INH. Compounds such as plumbagin and clofazimine, capable of generating superoxide anions, strengthen the activity of INH, contributing to a reduction of its inhibitory concentration (Bultovic et al. 2002). These observations support the suggested epistatic interaction between the KatG(S315T) mutation and mutations at the 5′-UTR region of the sodA gene.

Previous studies have also demonstrated that mutations within regulatory regions, such as the promoter of the inhA gene, lead to increased transcription of *inhA* mRNA, resulting in INH resistance (Vilchèze et al. 2006).

A previous large-scale analysis of whole genomes of *M. tuberculosis* (Billows et al. 2024) identified candidate mutations that may compensate for the fitness cost of INH-resistance mutations that downregulate KatG by increasing the expression of the gene ahpC, which codes for a protein with an enzymatic function similar to that of SodA (Sherman et al. 1996, 1999). In these works, Sherman et al. refuted a direct role for AhpC in detoxifying INH, but identified several strains of *M. tuberculosis* that were resistant to INH due to deficient activity of KatG and also overexpressed AhpC. They proposed that the corresponding mutations at the ahpC promoter are compensatory mutations selected from KatG-deficient, INH-resistant *M. tuberculosis* for mitigating their increased susceptibility to oxidative stress. However, these studies did not identify putative compensatory mutations in the gene sodA, and we did not identify any mutation in the gene ahpC in our data. Our results suggest that the same mechanism identified by Sherman et al. for ahpC act also on other genes that respond to oxidative stress such as sodA.

Our data also contain the clinical classification of isolates into two types: pulmonary TB (73 samples) and extra-pulmonary TB (72 samples). We investigated whether the strains that contain the KatG(S315T) mutation without the possible compensatory mutation at the 5′-UTR region of the gene sodA, which our results suggest are more susceptible to oxidative stress, are less likely to affect the lungs and cause the pulmonary type of TB. However, we could not differentiate the incidence of pulmonary TB according to the genotype KatG(S315T) with or without secondary mutations at the sodA gene, which rejects our hypothesis.

Interestingly, the inferred history of resistance mutations represented in Fig. 3 shows that all sequences of lineage 2 of *M. tuberculosis* (the Beijing lineage) in our dataset were descendants of an ancestral sequence that carried the resistance mutation KatG(S315T) and one mutation at the 5′-UTR region of sodA. Sequences of lineage 2 not carrying these mutations did not leave any offspring in our sample of 145 sequences isolated in Russian hospitals, which suggests that they have a strong selective disadvantage.

### The REGMLAME Score for Ranking Inferred Trees

Finally, we would like to mention some methodological aspects of the phylogenetic inference carried out in this work. By adopting different datasets (amino acids, DNA, and their combination, considering or removing synonymous and nonsynonymous mutations) and different substitution models, we verified that the inferred phylogenetic tree does not only depend on the chosen genomic sites and substitution model, as it is known but it also depends on the number of invariant sites that are considered. These invariant sites influence the fit of the parameter of the gamma distribution, and determine a large RF topological distance (0.33) between two phylogenetic trees inferred with different invariant sites.

We observed a very strong correlation between the LL score and the mean-fitted branch lengths across different tree topologies evaluated with the same model and data (*r* ≤ −0.95; Fig. 1). This indicates that the LL score depends almost exclusively on the mean branch length (at least for trees with good enough quality: the trees determined by excluding sites with nonsynonymous mutations present LL despite having short branches; they are significant outliers of the above correlation and are very different from all the other trees). This strong correlation between the fitted parameters (branch lengths) and the score of the fit (LL) is a possible cause of the bias of the ML method (Kuhner and Felsenstein 1994). This suggests that it is strongly advisable to reduce the bias by regularizing the fit of branch lengths (Hoerl and Kennard 1970) by penalizing too small or too large values of branch lengths. We proposed such a regularized ML and minimum evolution (REGMLAME) criterion in a recent work (Lorca-Alonso et al. 2025). Even if it is currently not possible to fit phylogenetic trees by minimizing the REGMLAME score, we applied this score a posteriori in order to rank the ML trees inferred with different substitution models. The REGMLAME score of a tree is consistent across different types of models (Fig. 2). We used the REGMLAME score for selecting 6 out of 16 phylogenetic tree topologies, and we verified that all qualitative results are robust across the selected trees.

## Conclusion

Our findings evidence a previously unknown weakness of the widespread resistance mutant KatG(S315T), which undergoes frequent and likely positively selected reversions, and suggest that compensatory mutations at the 5′-UTR region of the superoxide dismutase gene sodA may compensate the fitness loss due to the resistance mutation and enhance the reproductive rate. This scenario is similar to previous findings by Sherman et al. (1999) that identified compensatory mutations that overexpress AhpC, another protein that protects from oxidative stress, in KatG-deficient INH-resistant strains. ROS can act as an antimicrobial agent. Therapeutic options based on ROS are being investigated (Vaishampayan and Grohmann 2021). Therefore, we propose that combining INH treatment with ROS may be an effective way to limit the rise of resistance mutations such as KatG(S315T), to counteract these mutations when they are already present but not associated with compensatory mutations at genes involved in resistance to oxidative stress. This may be useful even in the presence of these compensatory mutations.

Alternatively, we think that it may also be worth investigating the design, or discovery from natural sources (Medema and van Wezel 2025), of a new prodrug (Lewis et al. 2024) similar to INH but that is activated by Sod3 instead of KatG.

## Methods

### Study Data

We considered 145 whole-genome sequences of *M. tuberculosis* strains isolated at Russian hospitals (Chernyaeva et al. 2021). We studied four sets of molecular sites: (i) 6,458 polymorphic amino acid sites, excluding 7 known resistance mutations, which are likely to undergo strong positive selection. (ii) 11,640 amino acid sites, including the previous set plus 5,182 invariant sites at which synonymous mutations occurred; (iii) 13,332 polymorphic DNA sites, either synonymous, nonsynonymous, or intergenic, excluding 7 sites with known resistance mutations; (iv) 6,874 DNA sites that do not modify amino acids since their mutations are synonymous or they are located at intergenic regions.

### Phylogenetic Analysis

For both amino acid and nucleotide data, we determined the first- and second-ranking substitution model with the program ModelTest-NG (Darriba et al. 2020) under the BIC (Schwarz 1978). The BIC penalizes the number of fitted parameters more strongly than alternative criteria: BIC=−2ln(LogLikelihood)+pln(n), where *p* is the number of fitted parameters and *n* is the number of columns in the multiple sequence alignment. This criterion was found to be optimal on simulated datasets (Luo et al. 2010).

With the program RAxML-NG (Kozlov et al. 2019), we determined the ML phylogenetic tree using 16 different combinations of partitioned datasets and models. We compared the topologies of the 16 inferred phylogenetic trees with the RF topological distance (Robinson and Foulds 1981) using the program Ktreedist (https://molevol-ibe.csic.es/Ktreedist.html, Soria-Carrasco et al. 2007).

We analyzed the six optimal trees according to the REGMLAME score (Lorca-Alonso et al. 2025), which regularizes the LL with the value expected given the mean branch length of the tree. For the six selected trees, we reconstructed ancestral sequences at the internal nodes of the tree using the option –ancestral of RAxML-NG, we counted the new mutation events for various classes of sites using an in-house script, and we tested whether the qualitative results were robust for all six selected phylogenetic trees.

### Analysis of Protein Thermal Dynamics with the TNM

As all ENMs, the TNM adopts a structure-based model of the energy of the native state, assuming that all the native interactions (identified as pairs of residues closer than a threshold) are minimally frustrated, i.e. the energy is minimal at the native interatomic distance. The TNM uses as degrees of freedom only the torsion angles of the chain backbone and the rigid body degrees of freedom of the different chains except the first one. Moreover, ENMs assume that the atomic fluctuations in the native state are small and approximate the energy function as a quadratic function of the displacements (see supplementary Supplementary Material for details). This approximation allows computing all thermal averages of the relevant quantities analytically using the normal modes of the system. In particular, we use the normal modes to compute the dynamical couplings between residues that belong to the same or different functional sites (Alfayate et al. 2019).

We predicted the thermal dynamics in the native state of the wild-type KatG protein (PDB code: 2cca) and the S315T mutant (PDB code: 2ccd) with the TNM (Mendez and Bastolla 2010), which is an ENM (Tirion 1996; Atilgan et al. 2001) in the space of torsion angles.

## Supplementary Material

evaf120_Supplementary_Data

## Data Availability

The data generated in this study are available as Supplementary material. The program torsional network model is available at https://github.com/ugobas/tnm.
