# Supplementary material for: Fitness Effect of the Isoniazid Resistance Mutation S315T of the Catalase-Peroxidase Enzyme KatG of Mycobacterium tuberculosis
Source: Genome Biol Evol. 2025 Jun 23;17(7):evaf120. doi: 10.1093/gbe/evaf120 (PMC12242383; doi:10.1093/gbe/evaf120)
Supplement: evaf120_Supplementary_Data [file evaf120_supplementary_data.zip › SuppMat_Tuberculosis_Tree.pdf]

Supplementary Figures for the paper: Fitness effect of the isoniazid resistance mutation S315T of the catalase-peroxidase enzyme KatG of Mycobacterium tuberculosis

Supplementary methods: Analysis of protein thermal dynamics with the torsional network model

As all ENMs, the TNM model adopts a structure-based model of the energy of the native state, assuming that all the native interactions (identified as pairs of residues closer than a threshold) are minimally frustrated, i.e. the energy is minimal at the native interatomic distance. It assumes that the force constant of the native interactions is the same for all residue types and decreases as a power law of the interatomic distance, and it approximates the energy at the second order assuming that the displacements from the native structure are small (harmonic approximation):

$$E_C = K \sum_{i<j} C_{ij} \frac{\left(r_{ij} - r_{ij}^{\mathrm{nat}}\right)^2}{\left(r_{ij}^{\mathrm{nat}}\right)^e}\,, \tag{1}$$

where C_{ij} is the binary contact matrix that represents the mean structure of the protein deposited in the PDB. $C_{ij} = 1$ for pairs of residues for which at least one pair of heavy atoms is closer than 4.5Å, otherwise if is zero. r_{ij} is the distance between the two closest atoms of residues i and j, r_{ij}^{nat} is its value in the native structure where the energy is minimal and $e = 6$ is an exponent that makes the force constant increase for closer residues. K is the scale parameter that may be individually adjusted for each protein by fitting the predicted fluctuations to the fluctuations observed in the B factor parameters (in this fit one has to consider rigid body motions, which strongly influence the B factors, Dehouck and Bastolla 2017).

The above features are similar in all ENMs, with the difference that the TNM considers the closest heavy atoms instead of the alpha carbon atoms as most ENMs do. The peculiarity of the TNM is that it considers as degrees of freedom the backbone torsion angles and the rigid body degrees of freedom of the different chains, and constraints all other degrees of freedom including bond angles and bond lengths. In this way, it has fewer degrees of freedom and faster computation than the ENMs that consider the three Cartesian degrees of freedom of the alpha carbons and it only represents the most physically relevant motions. Furthermore, the TNM constraints the torsion angles through quadratic potentials:

$$E = E_C + \kappa \sum_a \left(\varphi_a - \varphi_a^{\mathrm{nat}}\right)^2 \tag{2}$$

The model is very robust since it has only three global parameters $r_c = 4.5$Å, $e = 6$ and $\kappa = 0.2$, whose values were determined by comparing the predicted fluctuations through the fluctuations observed in experimental NMR ensembles. Since the energy function is quadratic (harmonic approximation), the equations of motion are solved by harmonic motions of the form $\varphi_a(t) = \varphi_a^\alpha \sin(\omega_\alpha t)$, where ω_α is the frequency of the normal mode α and φ_a^α is its amplitude vector. The normal modes constitute collective independent harmonic motions and allow reconstructing completely all protein motions, but only the most collective modes, which usually have low frequency, are physically relevant because they do not violate too much the harmonic approximation (Dehouck and Bastolla 2021). Using the normal modes, it is possible to compute analytically all the dynamical properties of the native state. The predicted fluctuations of the atomic coordinates correlate with fluctuations predicted with molecular dynamics and observed in the NMR ensemble. We use the normal modes to predict the dynamical couplings between residues that belong to same or different functional sites (Alfayate et al. 2019).

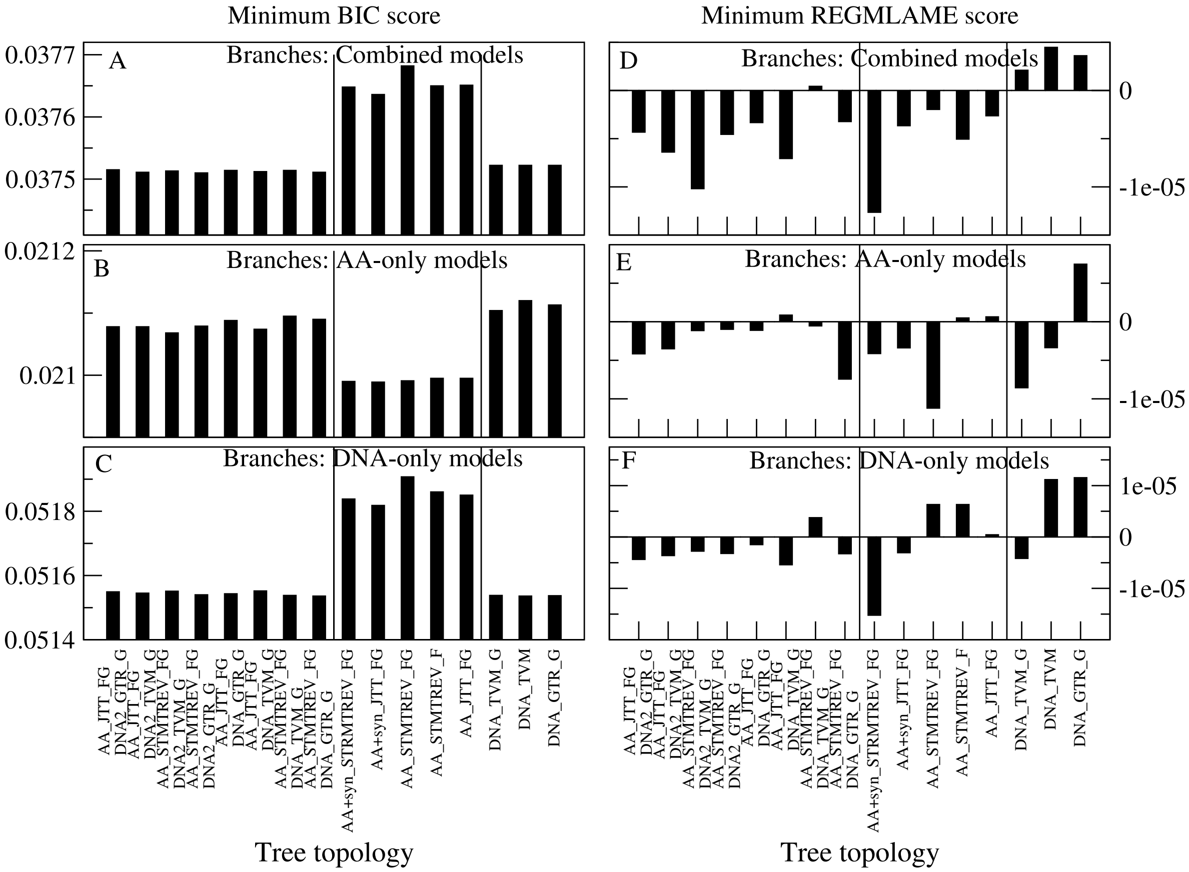

Figure S1: **Minimum values of the BIC scores and REGMLAME scores for each tree topology and different types of models.** For each tree topology on the horizontal axis, we show the minimum BIC score (plots A,B,C) and the minimum REGMLAME score (plots D,E,F) across the three types of substitution models that we considered: combined models (plots A and D), AA-only models (plots B and E) and DNA-only models (plots C and F). Trees inferred with combined models (left section of the plots) tend to have good (low) BIC scores also with DNA-only models (plot C). This is also true for trees inferred with DNA-only models (right section of the plots) with respect to combined models (plot A). In contrast, both kind of trees have poor (high) BIC scores with AA-only models (plot B). Trees inferred with AA-only models (mid section of the plots) have relatively poor BIC scores with other type of models (plots A and C). In contrast, some trees, in particular those inferred with combined models and AA-only models, have good (negative) REGMLAME scores for all types of models because the decrease in LL is compensated by the increase in branch lengths (plots D,E,F). In contrast, trees inferred with DNA-only models have poor (positive) REGMLAME score for at least one type of model.
