# Supplementary material for: Fitness Effect of the Isoniazid Resistance Mutation S315T of the Catalase-Peroxidase Enzyme KatG of Mycobacterium tuberculosis
Source: Genome Biol Evol. 2025 Jun 23;17(7):evaf120. doi: 10.1093/gbe/evaf120 (PMC12242383; doi:10.1093/gbe/evaf120)
Supplement: evaf120_Supplementary_Data [file evaf120_supplementary_data.zip › Supp_Table_S2_resistance_mutations.docx]

# 1) Known resistance mutations:

# Format: "gene-name_aawt-res-aamut (if we do not specify aawt-res-aamut is not specified, we consider all possible mutations # drug (if known))

"katG_S315T", # INH

"katG_E10G", "katG_C20S", "katG_D63E",

"katG_R104L", "katG_W107R", "katG_H108E", "katG_H108Q", "katG_A110V",

"katG_N138D", "katG_N138S", "katG_A139P", "katG_A139V", "katG_L148R",

"katG_Y229F", "katG_W300G",

"katG_T262R", "katG_H270Q", "katG_T275P", "katG_W328G",

"katG_Y337C", "katG_A350S",

"katG_S315G", "katG_S315I", "katG_S315N", "katG_S315R",

"katG_W321G", "katG_W321F", "katG_D381G",

"katG_L587M", "katG_L587P", "katG_L619P",

"katG_G629S", "katG_L634F", "katG_D735N", "katG_D735A",

"accD6_E224D",

"echa6", # cell wall biosynthesis

"efpA_E520V",

"eis", # KM

"embA", # EMB

"embB_M306V", "embB_M306I", # EMB

"embC", # EMB

"ethA", "ethR", # ETH

"fabD_A3T",

"fabG1", # fabG-inhA operon ETH

"fadE24_Q85R",

"fbpC",

"gidB", # STR

"gyrA",

"gyrB",

"inhA_I21T", "inhA_*15*", "inh_*8*", # INH

"iniB", # INH

"iniA_P3A", "iniA_R537H", "iniC_W83G",

"kasA", # INH?

"nat_T175A",

"ndh_R268H", "ndh_L104F", "ndh_E360K",

"pncA", # PZA

"rpoB_Q432K", # RMP, RIF

"rpsA", # STR SM

"rpsL", # STR SM

"rrs_*1401*", "rrs_*1402*", "rrs_*1484*","rrs_*1490*", # STR

"whiB7", # STR?

"Rv0340_V163I",

"Rv1592c_G9D", "Rv1592c_P42L", "Rv1592c_V430A",

"Rv1772_T4A"

# 2) Candidate secondary mutations that affect response to oxidative stress

# Format IG_gene1_N-or-C_gene2_C-or-N: intergenic region between N or C terminal of gene1 and N or C terminal of gene 2 # Comment

"ahpC", # Response to oxidative stress

"furA", # Negative regulator of katG

"oxyR'", # Positive regulator of katG. Inactivated in MTU

"sodA", # Superoxide dismutase; Destroys toxic superoxide anion radicals

"sodC", # Superoxide dismutase;

"IG_oxyR'_N_ahpc_N",

"IG_katG_N_furA_C",

"IG_furA_N_Rv1910c_C",

"IG_Rv3845_C_sodA_N",

"IG_Rv0431_C_sodC_C"
